# Impact of Comb Cell Diameter on Nectar Evaporation Efficiency in Honey Bees

**DOI:** 10.3390/insects16010071

**Published:** 2025-01-12

**Authors:** Shunhua Yang, Qingxin Meng, Tao Ye, Jianming Wang, Wenzheng Zhao, Yakai Tian, Kun Dong

**Affiliations:** Yunnan Provincial Engineering and Research Center for Sustainable Utilization of Honeybee Resources, Eastern Bee Research Institute, College of Animal Science and Technology, Yunnan Agricultural University, Kunming 650201, China; fengxue_20141011@163.com (S.Y.); 13015572330@163.com (Q.M.); rurosezwz@163.com (W.Z.)

**Keywords:** *Apis cerana*, *Apis mellifera*, comb cell, nectar evaporation, enzymatic activity

## Abstract

Honey bees transform nectar into honey through a combination of physical and chemical processes, with the physical process primarily involving the evaporation of excess water to concentrate the nectar. However, the factors affecting evaporation efficiency, such as evaporation duration, cell type, and bee species, remain incompletely understood. This study aimed to examine how these factors affect nectar evaporation efficiency during honey production. We measured the sucrose content in solutions subjected to combined active and passive evaporation, as well as passive evaporation alone. The results showed that eastern honey bee (EHB; *Apis cerana*) colonies were more efficient at concentrating sucrose solutions in worker cells than in drone cells under both combined active and passive evaporation conditions, as well as passive evaporation alone. Conversely, western honey bee (WHB; *Apis mellifera*) colonies exhibited greater efficiency in drone cells. Additionally, EHB colonies were more effective than WHB colonies in converting sucrose into fructose and glucose. Under passive evaporation, EHB colonies required at least 48 h to significantly concentrate the sucrose solution, while WHB colonies achieved similar concentrations in just 24 h. Sucrose content increased with the duration of passive evaporation. These findings provide insights into how honey bee colonies can efficiently produce mature honey during periods of abundant nectar flow.

## Simple Summary

A honey bee colony produces honey from nectar by removing excess water through evaporation in two phases: active evaporation on the bee’s tongue and passive evaporation in the honeycomb. The study evaluated the differences between eastern honey bee (EHB; *Apis cerana*) and western honey bee (WHB; *Apis mellifera*) colonies in nectar evaporation efficiency using western worker bee and drone bee comb cells. In our study, EHB and WHB did not show a preference for cell type (worker or drone cell) when storing nectar. With the increase in comb cell size, WHB colonies carried out the concentration more efficiently while the EHB exhibited a drop. However, due to high enzyme activity, EHB colonies showed more efficient conversion of sucrose into monosaccharides than the WHB colonies. Additionally, nectar concentration increased with the evaporation time in both cells. The efficiency of nectar concentration was higher for the WHB colonies than for the EHB colonies, probably due to their different ventilation strategies. Thus, we provide a foundation for beekeepers to develop effective strategies to achieve rapid honey production.

## 1. Introduction

Honey is formed from nectar or honeydew collected by bees or other social insects from living plants, created through the evaporation of water and enzymatic conversion by the insects [1]. Honey, a natural functional food [2], mainly consists of sugars and proteins, along with water. Fructose, glucose, and sucrose are the primary sugars in honey, while sucrase, amylase, catalase, phosphatase, and glucose oxidase are the main enzymes [3]. When nectar plants bloom and secrete nectar, forager bees collect it and carry it back to the hive, placing it in the comb cell as raw material for honey production. Nectar transforms into honey through physical and chemical processes facilitated by bees [4]. Physically, bees use hive temperature to evaporate excess water from nectar, while chemically, they add enzymes to hydrolyze sucrose into glucose and fructose, all within the comb cells [4,5]. Nectar is a crucial food source for bees and many other insects. Approximately 51 genera across six insect families exhibit nectar concentration behavior [6]. Bees concentrate nectar for six purposes: long-term storage, modifying nectar before mixing it with pollen provisions, removing excess water for efficient storage in the honey bees stomach, assisting with pollen transport, thermoregulation, and potentially in nest construction [6].

Young and middle-aged worker bees perform tasks inside the hive, such as cleaning cells, rearing brood, grooming, and processing nectar, while older worker bees forage outside [7,8,9]. This specialization in a bee colony is known as the division of labor, which enhances the efficiency of collective activities in a changing environment [10,11]. Extensive food transfer behavior occurs among worker bees within a colony [12], where food is passed directly from one worker bee to another, as well as to drones and the queen bee [13]. The food transferred is mainly nectar or honey regurgitated from the honey bees stomach and water but sometimes includes glandular secretions [14]. This food transfer likely plays a key role in maintaining colony cohesion [15]. The division of labor and food transfer in bees facilitate the rapid transformation of nectar into honey.

After collecting nectar from flowers, foraging bees do not store it directly in the hive [16,17]. Instead, they pass it to middle-aged bees, often called food processors, in the delivery area near the hive entrance [18,19,20,21]. Most processing bees pass the nectar to others on the way to the storage comb cells, often handing over most of the nectar [22,23,24,25]. The final nectar receiver deposits it in the comb cells for processing into honey, saving time and enabling foragers to harvest more efficiently [26]. Sucrose invertase is added as house bees pass nectar to each other and when it is transferred between cells [27,28]. This enzyme hydrolyzes sucrose into fructose and glucose, enabling the nectar to become honey in three to four days [29]. This initial nectar processing involves adding enzymes to achieve chemical reactions.

In the process of bees brewing honey from nectar, chemical reactions cause a qualitative change, transforming nectar into honey, while physical effects cause a quantitative change, concentrating low-concentration nectar into high-concentration nectar. The primary manifestation of the physical effect is nectar evaporation, powered by bees using thoracic muscle contractions to generate heat, maintaining the nest temperature at 33–36 °C [30,31,32,33]. Researchers have proposed the evaporation theory to describe the bees’ behavior of concentrating nectar, which involves two modes and three stages [34]. The first mode is active evaporation, where bees use their mouthparts to evaporate nectar. They repeatedly move their tongues to form a thin layer of nectar on the tongue’s surface, increasing the surface area and promoting rapid evaporation of excess water [35]. Active evaporation occurs both inside and outside the hive. Inside the hive, house bees repeatedly extend and retract their tongues, forming a thin nectar layer on the proboscis surface in nectar-filled cells, thereby stirring and increasing the nectar surface area to enhance evaporation [22,36]. Outside the hive, forager bees partially evaporate nectar while sucking it from flower nectaries and transporting it back to the hive. As forager bees move between flowers and return to the hive, they evaporate thin nectar layers on their tongues [37]. Bees that pre-concentrate nectar during foraging carry higher-concentration nectar in their stomachs, reducing the load and flight costs. The higher concentration also means less evaporation is needed to convert nectar into honey, thus saving honey processing costs [5].

At higher ambient temperatures, bees regulate head temperature by evaporating water from regurgitated nectar stored in their stomach. Heat affects bees physiologically through passive conduction and accelerated blood flow, which promotes hemolymph flow from the thorax to the head, leading to secondary stabilization of thorax temperature [38]. This mechanism enables flight at ultra-high ambient temperatures of 46 °C without overheating the head and thorax, despite the significant heat generated as a by-product of metabolism during flight [38,39]. In addition to cooling, this process also concentrates the thin nectar in the stomachs of forager bees [40]. Active evaporation is the first stage of water removal from nectar. In the second stage, bees use nest temperature [41] and ventilation [42] for passive evaporation to remove excess water from the nectar. This stage includes “painting” and “temporary hanging droplets”. Painting involves bees using their mouthparts as brushes to spread nectar onto the walls of empty cells, allowing it to flow down and cover most of the cell surface, thereby increasing the liquid nectar’s surface area for rapid water evaporation [22]. Temporary hanging of nectar droplets occurs when, during heavy nectar flow, the colony collects a large amount of nectar during the day and brings it back to the hive but lacks time to concentrate it immediately. As a result, house bees temporarily hang nectar droplets at the top of empty cells, focusing on active and passive evaporation of these droplets at night [22,43]. In the third stage, water evaporates directly from nectar stored in the main body of the cell, with evaporation occurring continuously [34,43].

Honey bee colonies rely on individual worker bees’ fanning behavior to promote air circulation within the hive. This behavior helps control temperature, humidity, and the concentration of respiratory gases [19,44,45,46,47]. On hot summer days, fanning bees can be observed near the entrance of natural or artificial hives, where they cling to supports with their legs and flap their wings to draw air out of the hive [48,49]. When ambient temperatures rise, foragers bring thin nectar and water into the hive. House bees use the evaporation of this nectar and water to regulate nest temperature [50,51], which depends on the circulating airflow maintained by fanning bees. The fanning behavior is influenced by the concentration of gases from bee respiration and metabolism. When carbon dioxide levels in the hive become too high, bees fan to exchange air inside and outside the hive [52,53,54]. The airflow generated by fanning bees not only reduces hive temperature [19] and increases humidity [36,55] but also facilitates air exchange [56,57]. Additionally, this airflow promotes the evaporation of nectar, accelerating honey ripening with increased ventilation [42,58].

The common feature of both active and passive evaporation of nectar by honey bee colonies is that the rapid evaporation of water from nectar primarily depends on hive temperature [41], nectar droplet surface area [22], and ventilation [42]. Higher hive temperatures, larger nectar droplet surface areas, and better ventilation increase the rate of water evaporation from nectar [43]. However, a greater volume of nectar stored in a cell or a higher initial nectar concentration reduces this rate [34,43]. Therefore, ample cell space and effective ventilation greatly enhance nectar concentration efficiency in honey bees. With a constant cell depth, larger cell diameters increase cell surface area, which effectively promotes water evaporation from nectar through “painting”, “temporary hanging droplets”, and “evaporation within the cell body”. The larger diameter of drone cells compared to worker cells [59] suggests that nectar evaporation efficiency may vary between the two cell types.

Some researchers have observed that western honey bees always flap their wings at the hive entrance, with their heads facing the hive interior and abdomens facing the exterior [60]. This causes airflow inside the hive to exit through the entrance [60], with water vapor also leaving through the entrance to the outside environment [61]. However, eastern honey bees flap their wings at the hive entrance, with their heads facing the exterior and abdomens facing the interior [62,63]. Thus, external air flows into the hive from the entrance, and water vapor may exit through the hive’s top vent.

Our hypotheses are that (1) when nectar sources are abundant, the honeycomb serves as a tool for nectar evaporation, and the honey bee colony has no specific preference for comb-type (worker comb, drone comb); (2) when cell depth is constant, the evaporation rate of nectar stored in large-diameter drone cells differs from that in small-diameter worker cells; (3) different ventilation strategies between eastern honey bees (EHB) and western honey bees (WHB) result in varying efficiencies in nectar evaporation within comb cells; and (4) EHB and WHB use the same nectar as raw material, but interspecific differences exist in the monosaccharide content and enzyme activity of the honey they produce.

This study aims to evaluate the effect of cell diameter on nectar evaporation efficiency during the honey-brewing process of EHB and WHB, using western worker and drone bee cells. We provide a theoretical basis for honey bee colonies to rapidly produce ripe honey from the perspective of comb cell type and bee species and offer a reference for beekeepers to develop scientific and reasonable feeding and management strategies to achieve rapid honey production during heavy nectar flow periods.

To verify these hypotheses, under active and passive evaporation, we (1) compared the storage capacity of sucrose solution in worker bee and drone bee combs within 24 h by the experimental colonies; (2) compared the total sugar content of sugar solutions processed by the experimental colonies for 48 h in different cell types; and (3) compared interspecies differences in monosaccharide content and enzyme activity in sugar solutions processed by the experimental colonies for 48 h. Under passive evaporation, we (4) compared sucrose content in different cell types during the same evaporation time; (5) compared sucrose content in the same cell types during different evaporation times, and (6) compared sucrose content in the same cell types evaporated by different bee species.

## 2. Materials and Methods

### 2.1. Active Evaporation Combined with Passive Evaporation

#### 2.1.1. Establishing the Experimental Honey Bee Colonies

Twenty experimental colonies were established in standard movable-frame Langstroth beehives with similar colony strength. The nest of each colony contained seven combs, all covered with adult worker bees. The queen bees were of similar age, and the worker bees were healthy and disease-free. The experimental colonies included ten EHB (*Apis cerana*) and ten WHB (*Apis mellifera*) colonies. During the summer months, when nectar sources were scarce, empty combs and uncapped honey combs were removed, leaving only three brood combs and two capped honey combs in the hive.

A newly built western worker bee comb and a newly built western drone bee comb were added to each experimental colony’s hive. The initial weight of the newly built comb was M_1_. These combs were placed symmetrically between the left and right walls of the hive, with a central comb serving as both an isolation barrier and the axis of symmetry. The combs used in this study were all constructed by WHB colonies using Langstroth standard movable frames with wax comb foundations. The cell base diameter of the worker bee comb foundation was 5.34 ± 0.0031 mm, and that of the drone bee comb foundation was 6.38 ± 0.0054 mm. The diameter and depth of the cells were measured following the method described by Yang et al. [64].

#### 2.1.2. Sucrose Solution Feeding to Experimental Bee Colonies

After adding the combs to the hives, each experimental colony was fed 2 kg of sucrose solution with 60% sucrose content (prepared by dissolving 60 g of sucrose in 40 g of water, mass ratio 6:4) in the evening. All colonies were provided with identical plastic feeders of the same type and size. After 24 h, the experimental comb was quickly removed from the hive and weighed as M_2_. The weight of the sucrose solution stored by the bees in the comb was calculated as m = M_2_ − M_1_. The comb was then returned to its original position in the hive, and the entire procedure was completed within 30 min.

#### 2.1.3. Measurement of Sugar and Sucrose Content in 48-h Processed Solutions by Experimental Bee Colonies

After experimental colonies were fed sucrose solution for 48 h, combs were removed from the hives. A Pasteur pipette was used to extract the sugar solution from worker and drone cells for total sugar content measurement using a refractometer. After measuring the total sugar content, the sugar solution was separated from all combs using a honey extractor. Worker and drone combs taken from EHB or WHB colonies were mixed to uniformly separate the sugar solution. However, combs taken from different species were not mixed for the sugar solutions, meaning that worker and drone combs from different species were processed separately. The 48-h sugar solution was used for analysis of monosaccharide, sucrose content, and enzyme activity.

#### 2.1.4. Analysis of Sugar Content and Enzyme Activity

Starting from feeding the colonies a sucrose solution containing 60% sucrose, bees processed the solution for 48 h through active and passive evaporation. Sucrose was not completely converted into monosaccharides. Therefore, the fructose, glucose, and sucrose content of the separated 48-h sugar solution, as well as the activities of sucrose convertase, amylase, catalase, phosphatase, and glucose oxidase, were also analyzed.

##### Analytical Methods for Sugar Content

Reagents: The standard samples for fructose, glucose, and sucrose were D-fructose (purity: 98.30%), D (+)-anhydrous glucose (purity: 99.40%), and sucrose (purity: 99.98%), respectively.

Drawing the standard curve: Fructose, glucose, and sucrose standards were prepared into a 100 mg/mL standard reserve aqueous solution and temporarily stored in a refrigerator at 4 °C. The standard reserve solution was then diluted with water into a series of standard solutions at 0.5, 1.0, 2.0, 5.0, 10, and 20 mg/mL for immediate use.

Sample extraction: A 100 mg sample was weighed, and 10 mL of pure water was added. The mixture was sonicated in a water bath for 30 min to dissolve thoroughly, passed through a 0.22 μm aqueous phase filter membrane, and prepared for machine testing.

Chromatographic conditions: Chromatographic column, Athena NH2-RP (II) HPLC (4.6 mm × 250 mm, 5 μm); column temperature, 40 °C; mobile phase, acetonitrile = 70:30; flow rate, 1.0 mL/min; injection volume, 10 μL; cell temperature, 0 °C; detector, differential refractive index detector; elution procedure, isocratic elution, with a duration of 35 min.

##### Analytical Methods for Enzyme Activity

Invertase: Invertase in honey quantitatively converts the substrate p-nitrophenyl-α-D-glucopyranoside to p-nitrophenol, and the p-nitrophenol content in the reaction system was determined by measuring absorbance at a specific wavelength [65].

Procedure: A 200 mg sample was taken, and 0.5 mL phosphate buffer was added, shaken to dissolve, and distilled water was added to make the total volume 0.75 mL for testing. A 250 μL p-nitrophenyl-α-D-glucopyranoside solution was preheated in a 40 °C water bath for 5 min. Then, 25 μL of the test solution was added, mixed rapidly, and reacted in a 40 °C water bath for 20 min. Afterward, 25 μL of termination solution (363.4 g trihydroxymethylaminomethane dissolved in 900 mL of water, pH adjusted to 9.5 with 3 mol/L HCl, diluted with water to 1 L) was added, vortexed, and cooled to room temperature, and the absorbance was measured at 400 nm. The control tube received the termination solution before the test solution, the standard tube received the standard solution instead of the substrate and sample, the blank tube received water instead of the substrate and sample, and the others were operated as the testing tube. Results were recorded as A_testing_, A_control_, A_standard_, and A_blank_. Calculations: ΔA_testing_ = A_testing_ − A_control_, ΔA_standard_ = A_standard_ − A_blank_. Each sample required a control tube, while blank and standard tubes needed to be tested 1–2 times.

Calculations and definition of units: 1 nmol of p-nitrophenol produced per minute per 1 g of honey was defined as an active unit of invertase.

$$\mathrm{Activity}\;\mathrm{of}\;\mathrm{invertase}\;(U/g)=\frac{△A_{\mathrm{testing}}\;}{△A_{\mathrm{standrad}}}\times \frac{V_{\mathrm{reaction}\;\mathrm{total}}\times V_{\mathrm{total}\;\mathrm{sample}}\times C_{\mathrm{standard}}}{△A_{\mathrm{standrad}}}\times \frac{F}{\mathrm{TW}}$$

Note that in the formula, C_standard_, the concentration of the standard solution (100 nmol/mL); V_reaction total_, the volume of the reaction system (0.275 mL); V_sample_, the added volume of the reaction system sample solution (0.025 mL); T, reaction time (20 min); F, sample dilution factor; V_total sample_, total volume of test solution (0.75 mL); W, sample weight in grams.

Amylase: Amylases are enzymes that hydrolyze starch and glycogen, primarily including α-amylase and β-amylase. Amylase catalyzes the hydrolysis of α-1,4 glucoside bonds in starch molecules to produce glucose, maltose, dextrin, and other substances. Iodine combines with unhydrolyzed starch to form a complex with an absorption peak at 660 nm. The active unit of amylase can be calculated based on this color reaction. Alpha-amylase is thermally stable, while β-amylase becomes inactive after a 15-min water bath incubation at 70 °C. Therefore, the activity of α-amylase was determined after the crude enzyme solution was heated at 70 °C for 15 min [66]. By measuring total amylase activity and subtracting it from α-amylase activity, the activity of β-amylase was calculated [67].

Procedure: A 200 mg sample was weighed, and 0.5 mL of distilled water was added and shaken to dissolve. Distilled water was then added to make the total volume 0.75 mL for testing.

Determination of total amylase: 0.1 mL of supernatant from the test solution was taken, and 0.1 mL of starch solution was added and mixed well. After reacting at 37 °C for 30 min, 0.1 mL of iodine solution and 0.2 mL of distilled water were added and mixed, and the absorbance was measured at 660 nm, recorded as A_total_.

Determination of Alpha-amylase: 0.1 mL of supernatant was taken after a 15-min water bath incubation at 70 °C. Then, 0.1 mL of starch solution was added, mixed, and reacted at 37 °C for 30 min. Afterward, 0.1 mL of iodine solution and 0.2 mL of distilled water were added and mixed, and the absorbance was measured at 660 nm, recorded as A_α_. Distilled water was used instead of the sample as a blank, and the absorbance was denoted as A_blank_.

Calculation and enzyme activity definition: One gram of honey reacting with starch at 37 °C for 30 min and hydrolyzing 1 mg of starch was defined as one active unit of amylase.

$$\mathrm{Total}\;\mathrm{amylase}\;\mathrm{activity}\;(U/g)=\frac{A_{\mathrm{blank}}-A_{\mathrm{total}}}{A_{\mathrm{blank}}}\times \frac{V_{\mathrm{starch}}\times V_{\mathrm{sample}}\times C_{\mathrm{starch}}}{V_{\mathrm{total}\;\mathrm{sample}}}\times \frac{30W}{T}$$

$$\alpha -\mathrm{amylase}\;\mathrm{activity}\;(U/g)=\frac{A_{\mathrm{blank}}-A_{\alpha }}{A_{\mathrm{blank}}}\times \frac{V_{\mathrm{starch}}\times V_{\mathrm{sample}}\times C_{\mathrm{starch}}}{V_{\mathrm{total}\;\mathrm{sample}}}\times \frac{30W}{T}$$

β-amylase activity (U/g) = total amylase activity (U/g) − α-amylase activity (U/g)

Note that in the formula, C _starch_ = 0.4 mg/mL (starch solution concentration); V_starch_ = 0.1 mL (volume of starch solution added); T = 30 min (reaction time); V_sample_ = 0.1 mL (volume of sample solution added); W = sample weight in grams; V_total sample_ = 0.75 mL (total volume of test solution).

Catalase: H_2_O_2_ reacts with ammonium molybdate to form a stable yellow complex, with a strong absorption peak at 405 nm. The absorbance value is proportional to the hydrogen peroxide concentration. By measuring the remaining H_2_O_2_ in the reaction system, the amount catalyzed by catalase was determined, reflecting catalase activity [68].

Procedure: A 200 mg sample was weighed, 1 mL of phosphate buffered saline (PBS) solution (pH 7.8) was added, and the mixture was shaken well to dissolve. Distilled water was added to bring the total volume to 1.25 mL for testing.

Twenty microliters of the test solution was placed in a 1.5 mL Ep tube, 100 μL of 20 μmol/mL H_2_O_2_ standard solution was added, and the mixture was well mixed. After a 10-min water bath incubation at 25 °C, 180 μL of 50 mmol/L ammonium molybdate tetrahydrate solution was added, mixed well, and left at room temperature for 10 min.

Two hundred microliters of the reaction solution was transferred to a 96-well plate, and the absorbance at 405 nm was measured, denoted as A testing. In the control tube, distilled water replaced the H_2_O_2_ standard solution; in the standard tube, PBS replaced the sample supernatant; and in the blank tube, both PBS and distilled water replaced the sample supernatant and H_2_O_2_ standard solution, respectively. The absorbance at 405 nm was measured using the same method and recorded as A _control_, A_standard_, and A_blank_, respectively. ΔA _testing_ = A_testing_ − A _control_, ΔA_standard_ = A_standard_ − A_blank_, ΔA = ΔA_standard_ − ΔA_testing_. Each sample required a control tube, while blank and standard tubes were tested 1–2 times.

Calculation and definition of unit: At 25 °C, the decomposition of 1 μmol H_2_O_2_ catalyzed by 1 g honey per hour was defined as an active unit of the enzyme.

$$\mathrm{catalase}\;\mathrm{activity}\;(U/g)=\frac{△A\;}{△A_{\mathrm{standrad}}}\times \frac{V_{\mathrm{standard}}\times V_{\mathrm{total}\;\mathrm{sample}}\times C_{\mathrm{standard}}}{V_{\mathrm{sample}}}\times \frac{F}{\mathrm{TW}}$$

Note that in the formula, C_standard_, concentration of standard samples (20 μmol/mL); V_standard_, the volume of added standard solution (0.1 mL); V_sample_, the volume of added sample solution (0.02 mL); T, reaction time (10 min); F, sample dilution factor; V_total sample_, total volume of test solution (1.25 mL); W, weight of the sample in grams.

Fructose-1,6-diphosphatase: Fructose-1,6-diphosphatase catalyzes 1,6-diphosphate fructose and water to produce 6-phosphate fructose and inorganic phosphorus. The addition of phosphogluconate isomerase and 6-phosphate glucose dehydrogenase to the reaction system produces 6-phosphate gluconate and NADPH under catalytic action. The rate of NADPH increase was measured at 340 nm to calculate fructose-1,6-diphosphatase activity [69].

Procedure: A sample of 200 mg was weighed, and 0.15 mL of distilled water was added. The solution was shaken to dissolve, and distilled water was added again to bring the total volume to 0.5 mL for testing. A 20 μL aliquot of the test solution was transferred into a 96-well plate. Then, 10 μL of glucose sulfate isomerase, 10 μL of 6-phosphate glucose dehydrogenase, and 160 μL of 1,6-diphosphate fructose were added in sequence. The mixture was immediately mixed, and timing was started upon the addition of the last reagent. The absorbance value A_1_ was recorded after 1 min of reaction, and A_2_ was recorded after 6 min of reaction at a wavelength of 340 nm. ΔA = A_2_ − A_1_ was calculated.

Calculation and definition of units: 1 nmol of NADPH produced per min per 1 g of honey was defined as an active unit of the enzyme.

$$\mathrm{The}\;\mathrm{activity}\;\mathrm{of}\;\mathrm{fructose}-1,6-\mathrm{diphosphatase}\;(\mathrm{nmol}/\min/g)=\;\frac{△A\times 10^{9}}{\varepsilon \times d}\times \frac{V_{\mathrm{reaction}\;\mathrm{total}}\times V_{\mathrm{total}\;\mathrm{sample}}}{V_{\mathrm{sample}}}\times \frac{F}{\mathrm{TW}}$$

In the formula, V_reaction volume_ is the reaction system volume (1 mL); *ε* is the NADPH molar extinction coefficient (6.22 × 10^3^ L/mol/cm); d is the 96-well plate optical diameter (0.5 cm); V_sample_ is the sample volume (0.02 mL); T is the reaction time (5 min); F is the sample dilution factor; V_total sample_ is the total volume of the test solution (0.5 mL), and W is the sample mass in grams.

Glucose-6-phosphatase: Glucose-6-phosphatase catalyzes the conversion of glucose-6-phosphate to glucose and inorganic phosphorus. The activity of glucose-6-phosphatase was measured using the molybdenum blue method by determining the increase in inorganic phosphorus content [70].

Procedure: A 200 mg sample was weighed, and 0.5 mL of phosphate buffer solution was added. The mixture was shaken well to dissolve, and distilled water was added to reach a total volume of 0.75 mL for testing.

A 20 μL aliquot of the test solution was taken, and 80 μL of 10 mmol/L glucose-6-phosphate was added. The mixture was reacted in a 25 °C water bath for 10 min and then placed immediately in a boiling water bath for 10 min. The sample was centrifuged at 10,000 rpm for 10 min, and 20 μL of the supernatant was taken. To this, 100 μL of phosphorus fixing reagent and 80 μL of distilled water were added, and the mixture was reacted in a 40 °C water bath for 10 min. The absorbance was then measured at a wavelength of 660 nm. To the control tube, 10 mmol/L glucose-6-phosphate was added after boiling in the water bath, and the same procedure as the test tube was followed. In a standard tube, 20 μL of 0.625 μmol/L phosphorus standard solution was taken, and 100 μL of phosphorus fixing reagent and 80 μL of distilled water were added. The mixture was reacted in a 40 °C water bath for 10 min, and absorbance was measured at a wavelength of 660 nm. In the blank tube, water was used instead of the phosphorus standard solution, with all other steps remaining the same. These were referred to as A_testing_, A _control_, A_standard_, and A_blank_. ΔA_testing_ =A_testing_ − A_control_, Δ A_standard_ =A_standard_ − A_blank_. Each sample required a control tube, while blank and standard tubes were tested 1–2 times.

Calculation and definition of enzyme unit: One nmol of inorganic phosphorus produced per 1 g of honey per min was defined as an active unit of the enzyme.

$$\mathrm{glucose}-6-\mathrm{phosphate}\;\mathrm{activity}\;(U/g)=\frac{△A\;}{△A_{\mathrm{standrad}}}\times \frac{V_{\mathrm{enzyme}\;\mathrm{catalyzed}}\times V_{\mathrm{test}\;\mathrm{solution}}\times C_{\mathrm{standard}}}{V_{\mathrm{sample}}}\times \frac{1000F}{\mathrm{TW}}$$

Note in the formula, C_standard_, the concentration of the standard solution (0.625 μmol/mL); V_enzyme-catalyzed_, the total volume of enzyme-catalyzed reaction (0.1 mL); V_test solution_, total volume of the added test solution (0.75 mL); T, reaction time (10 min); F, sample dilution factor; V _sample_, the volume of the added sample (0.02 mL); W, sample mass in grams. Unit conversion: 1 μmol = 1000 nmol.

Glucose oxidase: The concentration of H_2_O_2_ produced by glucose oxidase catalyzing glucose was determined using indigo carmine fading spectrophotometry, and glucose oxidase activity was obtained through standard curve conversion [71].

Procedure: A 200 mg sample was weighed, and 0.2 mL of pH 5.2 sodium acetate buffer was added. The mixture was shaken well to dissolve, and distilled water was added to bring the total volume to 0.5 mL. The solution was diluted twice and placed on ice for testing. Next, 50 μL of 0.2 mol/L glucose solution was taken in a 1.5 mL centrifuge tube, and incubation was carried out at 37 °C in a water bath for 5 min. After incubation, 50 μL of the test solution was added, mixed well, and allowed to react for 30 min at 37 °C. Then, 125 μL of indigo cochineal solution and 475 μL of distilled water were added, mixed well, and boiled in a water bath for 10 min. After cooling, the absorbance was measured at 615 nm and recorded as A testing. Then, 100 μL of sodium acetate buffer was taken, and 125 μL of indigo cochineal solution and 475 μL of distilled water were added, mixed well, and boiled in a water bath for 10 min. After cooling, the absorbance was measured at 615 nm and recorded as A blank. ΔA _testing_ = A_blank_ − A_testing_.

Drawing of the standard curve: A 1 mg/mL hydrogen peroxide standard solution was diluted into a series of gradient solutions of 100, 80, 50, 25, 10, and 5 μg/mL. A volume of 100 μL of each standard solution was taken, and 125 μL of indigo cochineal solution and 475 μL of distilled water were added, mixed well, and boiled in a water bath for 10 min. After cooling, the absorbance was measured at 615 nm and recorded as A standard. A standard blank was prepared by replacing the standard solution with water, and the same operation was performed. The absorbance was recorded as A standard blank. ΔA _standard_ = A _standard blank_ − A _standard_. The standard curve equation between the ΔA _standard_ and the ΔA _testing_ concentration was established.

Calculation and definition of units: The amount of enzyme required to catalyze glucose to produce 1 μg of H_2_O_2_ per min per 1 g of honey at 37 °C was defined as an active unit of the enzyme.

$$\mathrm{glucose}\;\mathrm{oxidase}\;\mathrm{activity}\;(U/g)=\frac{V_{\mathrm{reaction}\;\mathrm{total}}\times V_{\mathrm{total}\;\mathrm{sample}}}{V_{\mathrm{sample}}}\times \frac{\mathrm{CF}}{\mathrm{TW}}$$

Note in the formula, the H_2_O_2_ concentration was calculated by substituting the absorbance value of the sample into the standard curve; V_reaction total_, volume of enzymatic reaction system (0.1 mL); V_sample_, volume of sample added (0.05 mL); V_total sample_, total volume of the added test solution (0.5 mL); T, reaction time (30 min); F, sample dilution factor; W, sample mass in grams.

### 2.2. Passive Evaporation

#### 2.2.1. Establishment of Experimental Honey Bee Colonies

Eighteen experimental colonies were established in standard movable-frame Langstroth beehives with similar colony strength. Each colony’s nest consisted of seven combs, all covered with adult worker bees. The queen bees were of similar age, and the worker bees were healthy and disease-free. Among the experimental colonies, ten were EHB (*Apis cerana*) and ten were WHB (*Apis mellifera*). During the summer months, when the external environment lacked nectar sources, empty combs and uncapped honey combs were removed from the hives, leaving only three brood combs and two capped honey combs.

#### 2.2.2. Measurement of Sugar Content in Experimental Combs

Thirty-six newly constructed combs (18 worker bee combs and 18 drone bee combs) were used in this study. All combs were built by WHB colonies using Langstroth standard movable frames with wax comb foundations. The cell base diameter of the worker bee comb foundation was 5.34 ± 0.0031 mm, and the cell base diameter of the drone bee comb foundation was 6.38 ± 0.0054 mm.

The experimental combs were placed on a horizontal surface so that the hexagonal cell mouths were parallel to it. In the center of one side of the comb, 500 clustered cells of the same depth were selected. A sucrose solution (prepared by dissolving 40 g of sucrose in 60 g of water, mass ratio 4:6) with a sucrose content of 40% was injected into these cells using a disposable syringe. Sucrose solution was used instead of nectar, and the fluid level in the cells was kept flush with the cell mouths. The mesh size of the queen oviposition controller was adjusted to prevent different bee castes from entering through the mesh, and the comb injected with sucrose solution was inserted into the queen oviposition controller. Each experimental hive contained two queen oviposition controllers—one with the worker comb and the other with the drone comb (both filled with sucrose solution). These controllers were positioned symmetrically between the left and right walls of the hive, with a central comb serving as both an isolation barrier and the axis of symmetry, forming a cohesive nest structure with the other combs.

**Figure 1:**
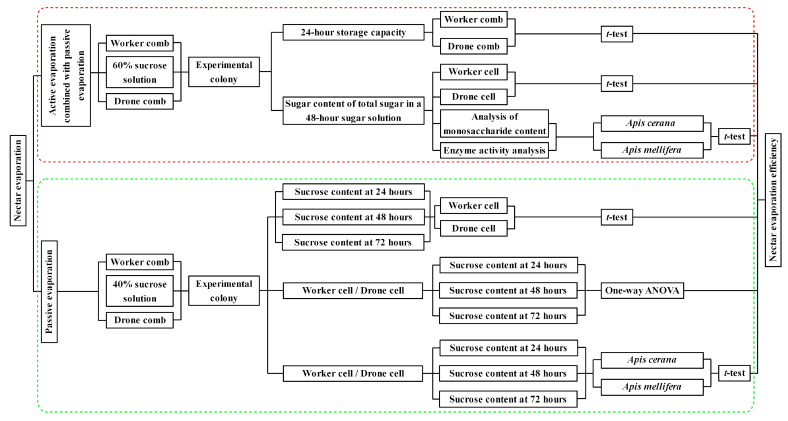
Experimental workflow. The red box outline the experimental process of active evaporation combined with passive evaporation, while the green box outline the experimental process of passive evaporation.

With the queen oviposition controller, bees in the hive could not enter the controller to contact the experimental comb. The sucrose solution in the comb cells underwent water evaporation due to nest temperature and ventilation, achieving passive evaporation. From the time the experimental combs were placed in the hive, they were removed every 24 h to measure the sucrose content until the experiment ended at 72 h. The combs were taken to the laboratory, where Pasteur pipettes were used to extract sucrose solution from the cells for measurement using a refractometer. Cells from which sucrose solution was extracted were not reused for subsequent measurements. During each measurement, 30 cells were randomly selected on each comb to measure the sucrose content. The combs were then returned to their original position in the hive, and the entire procedure was completed within 1 h. The experimental workflow is shown in Figure 1.

### 2.3. Statistical Analysis

Statistical analysis was conducted using GraphPad Prism 9.5 (GraphPad Software, San Diego, CA, USA). The Shapiro–Wilk test was used to assess normality for the cell size of combs, storage capacity of sucrose solution (m) in combs within 24 h, and the fructose content, glucose content, sucrose content, sucrose invertase, α-amylase, β-amylase, catalase, fructose-1, 6-diphosphatase, glucose-6-phosphatase, and glucose oxidase activities of the sugar solution processed by experimental bee colonies for 48 h. The Kolmogorov–Smirnov test was performed for other indexes. A significance level of alpha = 0.05 was applied; *p* > 0.05 indicated that the data conformed to a normal distribution.

An unpaired *t*-test was conducted for the cell size of combs, storage capacity of sucrose solution (m) in worker combs and drone combs within 24 h, the total sugar content in worker and drone comb cells processed by experimental colonies for 48 h, and the fructose content, glucose content, sucrose content, sucrose invertase, α-amylase, β-amylase, catalase, fructose-1, 6-diphosphatase, glucose-6-phosphatase, and glucose oxidase activities in sugar solution processed by EHB and WHB colonies for 48 h.

Under passive evaporation, an unpaired *t*-test was performed on the sucrose content of the sucrose solution in worker and drone comb cells processed by experimental colonies for 24, 48, or 72 h, as well as the sucrose content of the sucrose solution in worker or drone comb cells processed by EHB and WHB colonies for 24, 48, or 72 h. One-way ANOVA was conducted on the sucrose content in worker and drone cells among 24, 48, and 72 h. The post hoc Games–Howell test was used to determine the significance of differences between mean values, with a significance level of alpha = 0.05. All values are expressed as mean ± standard error.

## 3. Results

### 3.1. Cell Size

**Figure 2:**
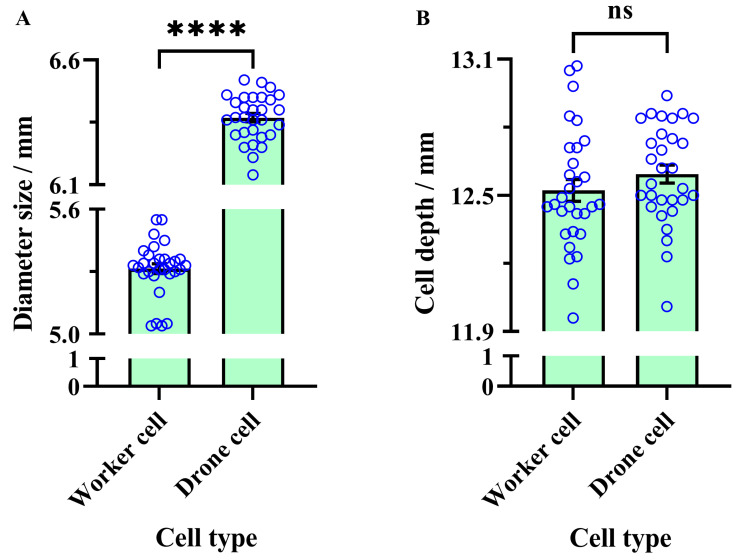
Comparison of cell dimensions in western honey bees. (**A**) Diameter comparison between worker and drone cells. (**B**) Depth comparison between worker and drone cells. The asterisks indicate significant differences (****, *p* < 0.0001), while “ns” denotes non-significant differences (*p* > 0.05). The blue circles are data values.

The average diameter of worker cells (5.31 ± 0.02 mm) was significantly smaller than that of drone cells (6.37 ± 0.02 mm) (unpaired *t*-test, t = 36.07, df = 58, *p* < 0.0001) (Figure 2A). In contrast, no significant difference was observed in the average depth of worker cells (12.52 ± 0.05 mm) compared to drone cells (12.59 ± 0.04 mm) (unpaired *t*-test, t = 1.145, df = 58, *p* = 0.257) (Figure 2B).

### 3.2. Active Evaporation Combined with Passive Evaporation

#### 3.2.1. Weight of Sucrose Solution Stored in Worker and Drone Combs Within 24 h

No significant difference was observed in the average weight of sucrose solution stored in western worker combs (485.0 ± 97.9 g) and western drone combs (493.0 ± 91.1 g) within 24 h by EHB colonies (unpaired *t*-test, t = 0.06, df = 18, *p* = 0.95).

Similarly, no significant difference was found in the average weight of sucrose solution stored in western worker combs (708.0 ± 44.1 g) and western drone combs (812.0 ± 41.1 g) over 24 h by WHB colonies (unpaired *t*-test, t = 1.73, df = 18, *p* = 0.10).

#### 3.2.2. Total Sugar Content of Sugar Solution Processed by Experimental Colonies in Different Cell Types for 48 h

**Figure 3:**
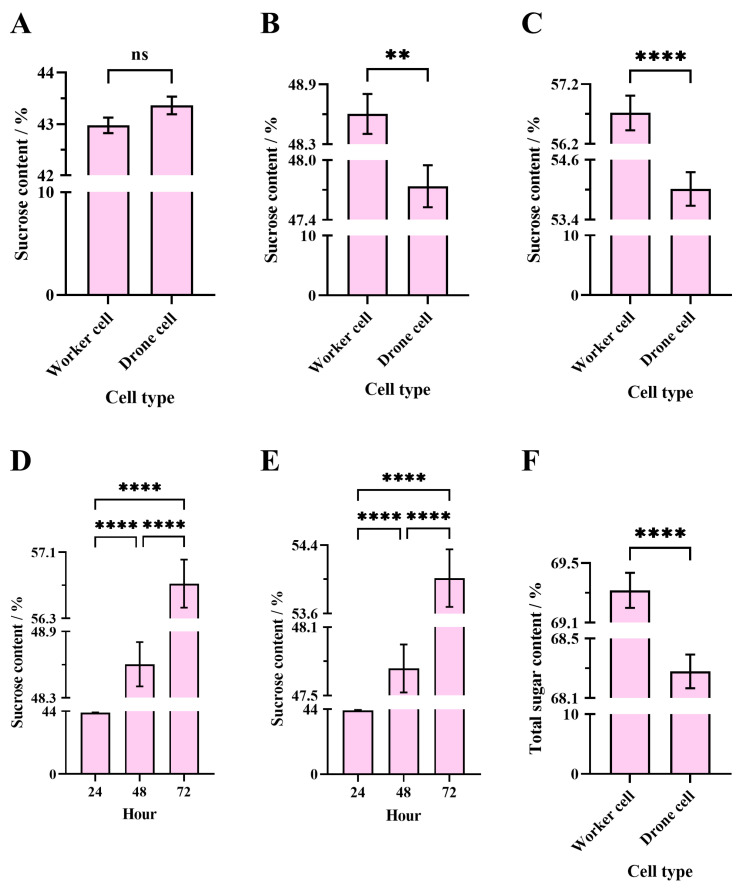
Analysis of concentrated sugar solutions in eastern honey bee colonies. (**A**–**C**) Comparison of the sugar content of sucrose solution in the worker and drone cells after 24, 48, and 72 h of passive evaporation, respectively. (**D**,**E**) Comparison of the sugar content of sucrose solution after 24, 48, and 72 h of passive evaporation between the worker and drone cells, respectively. (**F**) Comparison of the total sugar content of sucrose solution between the worker and drone cells after combined active and passive evaporation for 48 h. The asterisk indicates significant differences (**, *p* < 0.01; ****, *p* < 0.0001), while “ns” indicates not-significant differences (*p* > 0.05).

The average total sugar content of the solution processed for 48 h in western worker cells (69.32 ± 0.12%) was significantly higher than in western drone cells (68.28 ± 0.11%) by EHB colonies (unpaired *t*-test, t = 6.35, df = 478, *p* < 0.0001) (Figure 3F).

**Figure 4:**
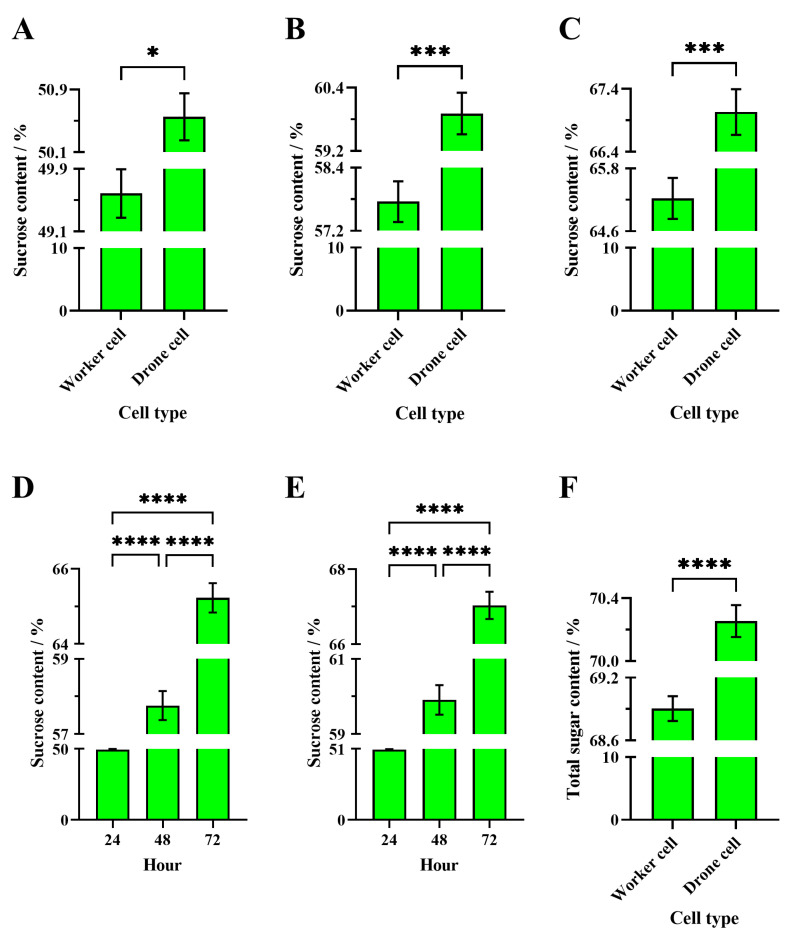
Analysis of concentrated sugar solutions in western honey bee colonies. (**A**–**C**) Comparison of the sugar content of sucrose solution in the worker and drone cells after 24, 48, and 72 h of passive evaporation, respectively. (**D**,**E**) Comparison of the sugar content of sucrose solution after 24, 48, and 72 h of passive evaporation between the worker and drone cells, respectively. (**F**) Comparison of the total sugar content of sucrose solution between the worker and drone cells after combined active and passive evaporation for 48 h. The asterisk indicates significant differences (*, *p* < 0.05; ***, *p* < 0.001; ****, *p* < 0.0001).

Conversely, the average total sugar content of the solution processed for 48 h in western worker cells (68.90 ± 0.12%) was significantly lower than in western drone cells (70.25 ± 0.10%) by WHB colonies (Welch’s *t*-test, t = 8.64, df = 467.9, *p* < 0.0001) (Figure 4F).

#### 3.2.3. Monosaccharide and Sucrose Content in Sugar Solution Processed by Experimental Bee Colonies for 48 h

**Figure 5:**
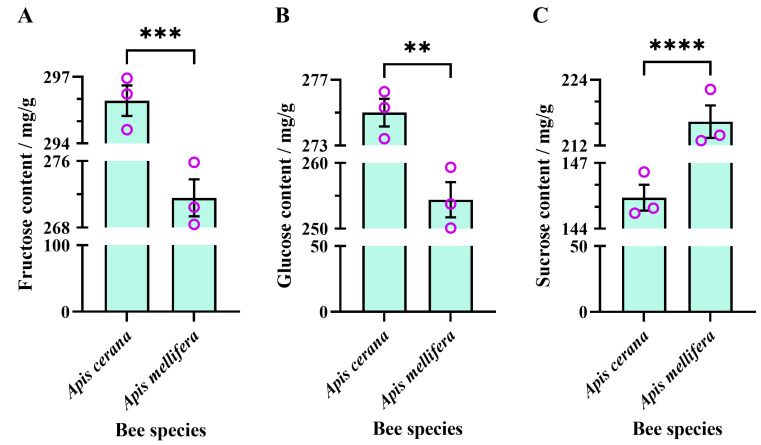
Interspecies comparison of monosaccharides and sucrose contents of sugar solutions processed by experimental bee colonies for 48 h under combined active and passive evaporation. (**A**) Fructose content, (**B**) glucose content, and (**C**) sucrose content. The asterisk indicates significant differences (**, *p* < 0.01; ***, *p* < 0.001; ****, *p* < 0.0001). The purple circles are data values.

The average fructose content of the sugar solution processed by EHB colonies for 48 h (295.90 ± 0.69 mg/g) was significantly higher than that processed by WHB colonies for 48 h (271.60 ± 2.22 mg/g) (unpaired *t*-test, t = 10.47, df = 4, *p* = 0.0005) (Figure 5A).

The average glucose content of the sugar solution processed by EHB colonies for 48 h (275.00 ± 0.84 mg/g) was significantly higher than that processed by WHB colonies for 48 h (254.40 ± 2.68 mg/g) (unpaired *t*-test, t = 7.33, df = 4, *p* = 0.0018) (Figure 5B).

The average sucrose content of the sugar solution processed by EHB colonies for 48 h (145.40 ± 0.59 mg/g) was significantly lower than that processed by WHB colonies for 48 h (216.40 ± 2.96 mg/g) (unpaired *t*-test, t = 23.48, df = 4, *p* < 0.0001) (Figure 5C).

#### 3.2.4. Enzyme Activity in Sugar Solution Processed by Experimental Bee Colonies for 48 h

**Figure 6:**
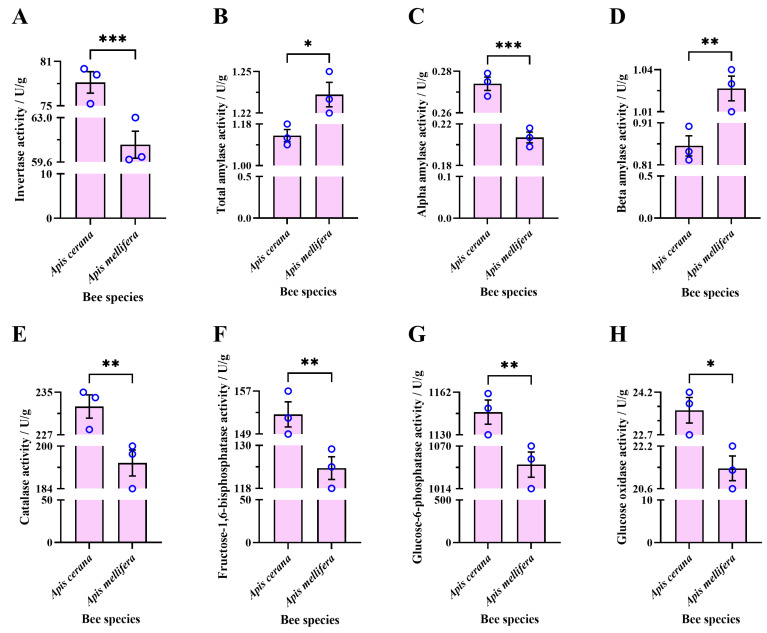
Interspecies comparison of enzymatic activities in the sugar solutions processed by experimental bee colonies for 48 h under combined active and passive evaporation. (**A**) Sucrose invertase, (**B**) total amylase, (**C**) alpha-amylase, (**D**) beta-amylase, (**E**) catalase, (**F**) fructose-1,6-diphosphatase, (**G**) glucose-6-phosphatase, and (**H**) glucose oxidase. The asterisk indicates significant differences (*, *p* < 0.05; **, *p* < 0.01; ***, *p* < 0.001). The blue circles are data values.

The average activity of sucrose invertase in the sugar solution processed by EHB colonies for 48 h (78.17 ± 1.45 U/g) was significantly higher than that processed by WHB colonies for 48 h (60.93 ± 1.04 U/g) (unpaired *t*-test, t = 9.67, df = 4, *p* = 0.0006) (Figure 6A).

The average activity of total amylase in the sugar solution processed by EHB colonies for 48 h (1.13 ± 0.03 U/g) was significantly lower than that processed by WHB colonies for 48 h (1.23 ± 0.01 U/g) (unpaired *t*-test, t = 3.71, df = 4, *p* = 0.0207) (Figure 6B).

The average activity of α-amylase in the sugar solution processed by EHB colonies for 48 h (0.27 ± 0.003 U/g) was significantly higher than that processed by WHB colonies for 48 h (0.21 ± 0.01 U/g) (unpaired *t*-test, t = 10.97, df = 4, *p* = 0.0004) (Figure 6C).

The average activity of β-amylase in the sugar solution processed by EHB colonies for 48 h (0.86 ± 0.02 U/g) was significantly lower than that processed by WHB colonies for 48 h (1.03 ± 0.01 U/g) (unpaired *t*-test, t = 6.69, df = 4, *p* = 0.0026) (Figure 6D).

The average activity of catalase in the sugar solution processed by EHB colonies for 48 h (232.3 ± 2.19 U/g) was significantly higher than that processed by WHB colonies for 48 h (193.7 ± 4.91 U/g) (unpaired *t*-test, t = 7.19, df = 4, *p* = 0.0020) (Figure 6E).

The average activity of fructose-1,6-diphosphatase in the sugar solution processed by EHB colonies for 48 h (152.7 ± 2.33 U/g) was significantly higher than that processed by WHB colonies for 48 h (123.7 ± 3.18 U/g) (unpaired *t*-test, t = 7.35, df = 4, *p* = 0.0018) (Figure 6F).

The average activity of glucose-6-phosphatase in the sugar solution processed by EHB colonies for 48 h (1147 ± 9.07 U/g) was significantly higher than that processed by WHB colonies for 48 h (1046 ± 16.58 U/g) (unpaired *t*-test, t = 5.36, df = 4, *p* = 0.0058) (Figure 6G).

The average activity of glucose oxidase in the sugar solution processed by EHB colonies for 48 h (23.57 ± 0.45 U/g) was significantly higher than that processed by WHB colonies for 48 h (21.37 ± 0.46 U/g) (unpaired *t*-test, t = 3.41, df = 4, *p* = 0.0270) (Figure 6H).

### 3.3. Passive Evaporation

#### 3.3.1. Sucrose Content in Different Cell Types for the Same Evaporation Time

##### Apis Cerana

After 24 h of passive evaporation by EHB colonies, no significant difference was observed in average sucrose content between sucrose solutions in WHB worker cells (42.97 ± 0.15%) and WHB drone cells (43.36 ± 0.17%) (Welch’s *t*-test, t = 1.70, df = 528.9, *p* = 0.0899) (Figure 3A).

After 48 h of passive evaporation by EHB colonies, the average sucrose content in WHB worker cells (48.60 ± 0.20%) was significantly higher than in WHB drone cells (47.74 ± 0.21%) (unpaired *t*-test, t = 2.99, df = 538, *p* = 0.003) (Figure 3B).

After 72 h of passive evaporation by EHB colonies, the average sucrose content in WHB worker cells (56.72 ± 0.29%) was significantly higher than in WHB drone cells (54.01 ± 0.34%) (Welch’s *t*-test, t = 6.08, df = 526.2, *p* < 0.0001) (Figure 3C).

##### Apis Mellifera

After 24 h of passive evaporation by WHB colonies, the average sucrose content in WHB worker cells (49.59 ± 0.31%) was significantly lower than in WHB drone cells (50.55 ± 0.30%) (unpaired *t*-test, t = 2.24, df = 538, *p* = 0.0253) (Figure 4A).

After 48 h of passive evaporation by WHB colonies, the average sucrose content in WHB worker cells (57.76 ± 0.39%) was significantly lower than in WHB drone cells (59.91 ± 0.39%) (unpaired *t*-test, t = 3.91, df = 538, *p* = 0.0001) (Figure 4B).

After 72 h of passive evaporation by WHB colonies, the average sucrose content in WHB worker cells (65.23 ± 0.39%) was significantly lower than in WHB drone cells (67.03 ± 0.36%) (unpaired *t*-test, t = 3.39, df = 538, *p* = 0.0008) (Figure 4C).

#### 3.3.2. Sucrose Content in Same Cell Types at Different Evaporation Times

##### Apis Cerana

The passive evaporation time for EHB colonies significantly affected the sucrose content of the sucrose solution in WHB worker bee cells (Welch’s ANOVA test, W = 945.4; df = 2507.7; *p* < 0.0001). Games–Howell multiple comparisons showed that the average sucrose content after 72 h of passive evaporation (56.72 ± 0.29%) was significantly higher than after 24 h (42.97 ± 0.15%, *p* < 0.0001) and 48 h (48.60 ± 0.20%, *p* < 0.0001). The 48-h sucrose content was also significantly higher than the 24-h sucrose content (*p* < 0.0001) (Figure 3D).

Similarly, passive evaporation time for EHB colonies significantly affected the sucrose content of the sucrose solution in WHB drone bee cells (Welch’s ANOVA test, W = 430.1; df = 2509.9; *p* < 0.0001). Games–Howell multiple comparisons showed that the average sucrose content after 72 h of passive evaporation (54.01 ± 0.34%) was significantly higher than after 24 h (43.36 ± 0.17%, *p* < 0.0001) and 48 h (47.74 ± 0.21%, *p* < 0.0001). The 48-h sucrose content was also significantly higher than the 24-h sucrose content (*p* < 0.0001) (Figure 3E).

##### Apis Mellifera

The passive evaporation time for WHB colonies significantly affected the sucrose content of the sucrose solution in WHB worker bee cells (Welch’s ANOVA test, W = 502.3; df = 2531.2; *p* < 0.0001). Games–Howell multiple comparisons showed that the average sucrose content after 72 h of passive evaporation (65.23 ± 0.39%) was significantly higher than after 24 h (49.59 ± 0.31%, *p* < 0.0001) and 48 h (57.76 ± 0.39%, *p* < 0.0001). The 48-h sucrose content was also significantly higher than the 24-h sucrose content (*p* < 0.0001) (Figure 4D).

Similarly, passive evaporation time for WHB colonies significantly affected the sucrose content of the sucrose solution in WHB drone bee cells (Welch’s ANOVA test, W = 629.5; df = 2530.4; *p* < 0.0001). Games–Howell multiple comparisons showed that the average sucrose content after 72 h of passive evaporation (67.03 ± 0.36%) was significantly higher than after 24 h (50.55 ± 0.30%, *p* < 0.0001) and 48 h (59.91 ± 0.39%, *p* < 0.0001). The 48-h sucrose content was also significantly higher than the 24-h sucrose content (*p* < 0.0001) (Figure 4E).

#### 3.3.3. Sucrose Content in Sucrose Solution Evaporated by Different Bee Species in the Same Cell Types

##### Worker Cell

**Figure 7:**
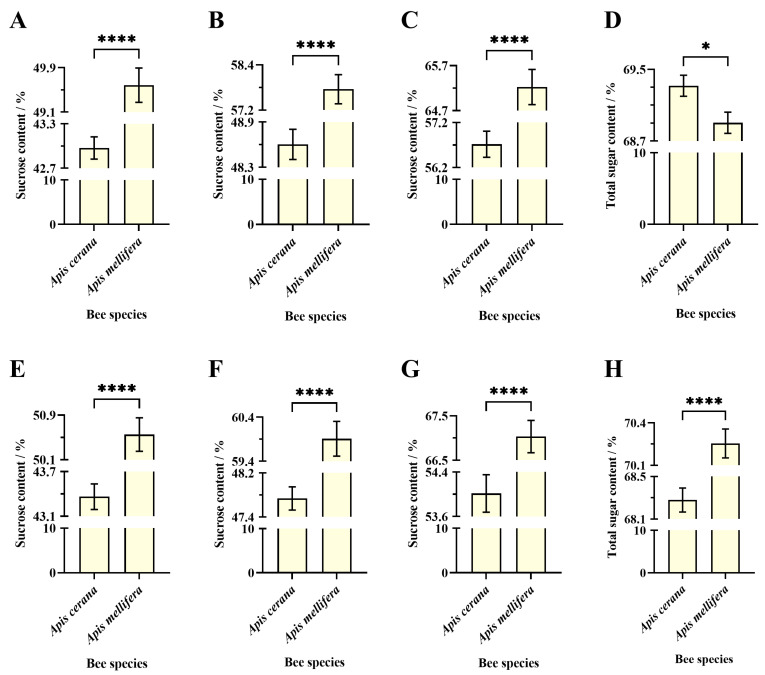
Interspecies comparison of the sugar content of sucrose solution concentrated by experimental bee colonies in worker and drone cells. (**A**–**C**) Interspecies comparisons of the sugar content of sucrose solution in worker cells after 24, 48, and 72 h of passive evaporation, respectively. (**D**) Interspecies comparison of sugar content of sucrose solution after 48 h of combined active and passive evaporation in worker cells. (**E**–**G**) Interspecies comparisons of the sugar content of sucrose solution after 24, 48, and 72 h of passive evaporation in drone cells, respectively. (**H**) Interspecies comparison of sugar content of sucrose solution after 48 h of combined active and passive evaporation in drone cells. The asterisk indicates significant differences (*, *p* < 0.05; ****, *p* < 0.0001).

The average sucrose content of the sucrose solution in Western worker bee cells after 24 h of passive evaporation by EHB colonies (42.97 ± 0.15%) was significantly lower than that after 24 h of passive evaporation by WHB colonies (49.59 ± 0.31%) (Welch’s *t*-test, t = 19.19, df = 389.8, *p* < 0.0001), as shown in Figure 7A.

After 48 h of passive evaporation, the average sucrose content in western worker bee cells processed by EHB colonies (48.60 ± 0.20%) remained significantly lower than that processed by WHB colonies (57.76 ± 0.39%) (Welch’s *t*-test, t = 21.03, df = 403.6, *p* < 0.0001) (Figure 7B).

Similarly, after 72 h of passive evaporation, the average sucrose content in western worker bee cells processed by EHB colonies (56.72 ± 0.29%) was significantly lower than that processed by WHB colonies (65.23 ± 0.39%) (Welch’s *t*-test, t = 17.48, df = 496.5, *p* < 0.0001) (Figure 7C).

After 48 h of active and passive evaporation, the total sugar content in the sucrose solution processed by EHB colonies (69.32 ± 0.12%) was significantly higher than that processed by WHB colonies (68.90 ± 0.12%) (unpaired *t*-test, t = 2.471, df = 478, *p* = 0.0138) (Figure 7D).

##### Drone Cell

The average sucrose content of the sucrose solution in western drone bee cells after 24 h of passive evaporation by EHB colonies (43.36 ± 0.17%) was significantly lower than that after 24 h of passive evaporation by WHB colonies (50.55 ± 0.30%) (Welch’s *t*-test, t = 20.81, df = 429.2, *p* < 0.0001) (Figure 7E).

After 48 h of passive evaporation, the average sucrose content in Western drone bee cells processed by EHB colonies (47.74 ± 0.21%) remained significantly lower than that processed by WHB colonies (59.91 ± 0.39%) (Welch’s *t*-test, t = 27.26, df = 410.5, *p* < 0.0001) (Figure 7F).

Similarly, after 72 h of passive evaporation, the average sucrose content in western drone bee cells processed by EHB colonies (54.01 ± 0.34%) was significantly lower than that processed by WHB colonies (67.03 ± 0.36%) (unpaired *t*-test, t = 26.27, df = 538, *p* < 0.0001) (Figure 7G).

After 48 h of active and passive evaporation, the total sugar content in the sucrose solution processed by EHB colonies (68.28 ± 0.11%) was significantly higher than that processed by WHB colonies (70.25 ± 0.10%) (Welch’s *t*-test, t = 12.96, df = 473, *p* < 0.0001) (Figure 7H).

## 4. Discussion

### 4.1. The Significance of Bees Storing Honey in the Drone Cells

Adult worker honey bees use wax secreted from abdominal glands to construct two types of hexagonal cells [72]. One is the smaller-diameter worker cell, used by the colony to rear worker bees and store honey and pollen [73]. The other is the larger-diameter drone cell, used to rear drones [59]. Western honey bees prefer storing honey in worker bee combs rather than in drone bee combs and rarely store pollen in drone combs [74]. Although the average area of drone combs in natural western honey bee colonies is only 17.0% of the total comb area, ranging from 10.0% to 24.2%, the average percentage of drone cells used for food storage is 8.2%, with a range of 1.0% to 16.0% [73], indicating that cave-dwelling bee species use drone cells to store honey under certain conditions. Additionally, open-air bee species, such as *Apis florea*, also store honey in drone cells [75]. After a colony abandons its nest, only a few nurse bees remain on the comb, and when the wax cap of the drone cells is removed, these cells are found to be full of honey [75]. Honey bees avoid storing food in drone combs because they fine-tune their hive structure by adaptively allocating drone combs between survival and reproduction. Worker bees adjust honey storage locations in the hive seasonally, optimizing the limited drone comb space. In spring and early summer, they preferentially remove honey from drone combs to rear drones, while in late summer and autumn, they do not, leading to a large number of drones in spring and fewer in late summer [76]. There was no significant difference in the weight of sucrose solution stored by experimental colonies in worker and drone combs within 24 h under active and passive evaporation. This suggests that when nectar is abundant, colonies do not show a clear preference for comb-type when storing nectar. During major nectar flows, beekeepers can use worker combs in the brood box to rear worker broods and add drone combs in the super box to store honey. The queen should be isolated with a queen excluder between the brood and super boxes to prevent her from laying eggs in the drone cells in the super box. The larger diameter of drone cells makes it easier to centrifuge out fully ripened, highly concentrated honey.

### 4.2. Difference in Nectar Evaporation Between Worker and Drone Cells

Whether under active or passive evaporation, the total sugar content in worker cells was always significantly higher than in drone cells after sucrose concentration by EHB colonies (except for 24-h passive evaporation). However, the total sugar content in worker cells was always significantly lower than in drone cells after sucrose concentration by WHB colonies. These results indicate that with constant cell depth, enlarging cell diameter to concentrate nectar reduces honey-making efficiency in EHB colonies but improves it in WHB colonies. This further suggests that nectar evaporation efficiency is influenced not only by cell diameter but also by bee species differences. The average diameter of Western honey bee worker and drone cells is 5.2 mm and 6.2 mm or 6.4 mm, respectively [73,77]. The drone cell diameter is clearly 1.0 or 1.2 mm larger than that of the worker cell. Therefore, with constant cell depth, both the surface area and volume of the cell increase as its diameter enlarges. This increase in surface area potentially enlarges the nectar droplet surface, promoting faster evaporation of excess water. Although honey bees prefer to store honey in worker cells rather than drone cells [74], natural honey bee combs contain specialized honey storage cells, which can reach a depth of 34 mm and a diameter of 9.2 mm [78]. The larger diameter of honey storage cells compared to drone cells indicates that bees tend to use larger cells for honey storage or brewing.

### 4.3. Difference in Concentrated Nectar Between EHB and WHB Colonies

Under passive evaporation, a 40% sucrose solution is concentrated in worker or drone cells for 24–72 h. The sucrose content in the solution concentrated by WHB colonies was consistently higher than in the solution concentrated by EHB colonies. This indicates that even with the same initial nectar concentration, cell type, and time frame, different bee species can produce significant concentration differences. Under active evaporation combined with passive evaporation, a 60% sucrose solution is concentrated in WHB worker cells for 48 h. The sucrose content in the solution concentrated by EHB colonies is consistently higher than that in WHB colonies. However, when the same 60% sucrose solution was concentrated in drone cells for 48 h, the sucrose content in the solution concentrated by EHB colonies was consistently lower than that in WHB colonies. This suggests that WHB generally have an advantage over EHB in nectar concentration, regardless of the method used.

This difference may result from the distinct ventilation strategies of EHB and WHB. WHB always flap their wings at the hive entrance with their heads facing inward and abdomens facing outward [60]. This allows fresh air to enter through vents at the top of the hive, while stale air and water vapor exit through the hive entrance [61]. In contrast, EHB flap their wings with abdomens facing inward and heads facing outward [62,63]. Fresh air enters through the hive entrance, and stale air, along with water vapor, exits through the top vents [57,79]. The lower nectar concentration brought back by forager bees increases hive humidity [55]. EHB keepers often observe water droplets on the inner hive walls, linked to low nectar concentration and their ventilation strategy. Bees prefer storing nectar at the top of combs [74,80]. The nectar at the top is concentrated first, leading to higher concentrations and hygroscopicity [34,81]. The fanning behavior of EHB results in outside air entering the hive through the entrance, while water vapor exits through the top vents. Consequently, vapor from the middle and bottom cells passes through the top cells, diluting the high-concentration nectar. Larger cell diameters accelerate water evaporation from low-concentration nectar, and the previously concentrated high-concentration nectar absorbs water more rapidly. This explains why larger cell diameters reduce EHB colonies’ efficiency in honey production.

### 4.4. Variability of Honey Concentration

Under passive evaporation, a sucrose solution with a 40% sucrose content, concentrated by bee colonies for 24–72 h, shows a significant increase in sucrose content as the concentration time increases, regardless of whether the solution is concentrated in worker or drone cells. When EHB colonies concentrated the sucrose solution for 24, 48, and 72 h in worker or drone cells, the sucrose content increased by 2.97% or 3.36%, 8.60% or 7.74%, and 16.72% or 14.01%, respectively. This indicates that for EHB colonies, at least 48 h are required to produce a significant concentration effect. Conversely, when WHB colonies concentrated the sucrose solution for 24, 48, and 72 h in worker or drone cells, the sucrose content increased by 9.59% or 10.55%, 17.76% or 19.91%, and 25.23% or 27.03%, respectively. Thus, WHB colonies only need 24 h to achieve a significant concentration effect. In beekeeping, the process of transforming nectar into honey has a moisture content limit [82]. The average moisture content of honey is 17.2%, ranging from 13.4% to 22.9% [82]. As nectar ripens, it becomes hygroscopic [34,81], meaning it can absorb water from the beehive atmosphere, leading to increased water content. As a result, honey in the hive maintains a dynamic balance between dilution and concentration, and honey with high water content is prone to fermentation [83,84].

The total sugar concentration in nectar varies widely, typically ranging from 20% to 40%, depending on plant species and environmental conditions [85,86]. Nectar transforms into honey through five stages [87]. The first stage is nectar secretion by the floral nectaries of honey plants. In the second stage, forager bees collect nectar from flower nectaries and temporarily store it in their honey stomachs [88]. The third stage involves house bees receiving nectar from forager bees and temporarily storing it in their honey stomachs [89]. In the fourth stage, the nectar becomes uncovered, unripened honey in the cells [90]. Finally, the fifth stage is capped honey, which is fully ripened and stored in cells [91]. The concentration of nectar or honey changes at each stage. The nectar’s initial concentration is influenced by the honey plant and environment, but from the second stage onward, it is entirely controlled by bees [87]. Diagnostic radioentomology reveals that within 48 h after foraging, bees return to the hive and transfer nectar to house bees; the house bees store carbohydrates in clusters of cells with similar sugar concentrations in a nonrandom pattern [92]. This behavior, indicated by patchy spatial cell distributions, accelerates nectar ripening by reducing the distance between cells with similar sugar concentrations [4,93]. As a result, colonies that optimize storage strategies gain an evolutionary advantage over less efficient colonies [92]. Honey bee colonies can only store nectar long-term by concentrating it and converting it into ripened honey. This process consumes 25% to 50% of the energy transported to the hive in the form of nectar [94]. The spatial pattern of early carbohydrate storage confines the tasks of honey ripening to specific nest areas, reducing energy expenditure. However, diagnostic radioentomology has shown that after three days of storage activity, cells with varying sugar concentrations begin to mix, and spatial clustering of similar sugar concentrations is only occasionally observed [93]. Thus, in the early stages of nectar storage, the clustering of cells by sugar concentration does not increase efficiency. Food-storing bees store carbohydrates randomly in the combs rather than searching for cells with matching sugar concentrations [95].

The transformation of nectar into honey by dehydration in the nest is a dynamic process characterized not only by the changing concentration but also by the shifting storage location [96]. Before final storage, nectar undergoes position migration within the nest, a necessary step in its conversion to honey [96]. Since the total sugar concentration of nectar or raw honey removed by bees from the cells is lower than that of stored nectar or ripened honey, removing and further manipulating low-concentration nectar or immature honey increases the evaporation rate of water [97]. Nectar’s sugar concentration varies across different locations within the same cell [98]. Computed tomography shows that nectar’s density and sugar concentration increase from the bottom to the mouth of the cell [4]. The sugar content of nectar at the bottom of the cell is 73.6% ± 2.1% and at the mouth is 91.4% ± 1.4% [4]. When house bees deposit nectar or raw honey into a target cell, if honey has already been deposited there, they immerse their mouthparts in the existing honey and directly add their honey droplets [22,99]. This might explain the varying concentrations of nectar or honey in different locations within the same cell, further indicating that honey is a substrate with uneven concentrations.

### 4.5. Monosaccharide Content and Enzyme Activity Differences in Honey Brewed by EHB and WHB Colonies

In the absence of external nectar sources, we fed the experimental colonies a 60% sucrose solution to sustain them and stored the sugar in newly built experimental combs. After 48 h, the combs containing the brewed sugar solution were removed and centrifuged. Testing revealed that the main sugars were fructose, glucose, and sucrose, while the primary enzymes were sucrose invertase, amylase, catalase, phosphatase, and glucose oxidase. This result indicates that the bees added enzymes to the sucrose solution, with invertase hydrolyzing the sucrose into fructose and glucose. Numerous studies have shown that honey contains invertases that hydrolyze sucrose [100,101,102,103,104,105], which are secreted from the hypopharyngeal gland of worker bees [106,107,108,109]. The main function of amylase in honey is to hydrolyze starch into maltose [110,111], reducing honey’s viscosity, which aids in separating honey from combs [112]. Amylase in honey comes from three sources: pollen [113,114], nectar [115,116], and the hypopharyngeal gland of worker bees [117,118,119,120]. In addition to the pharyngeal gland, the salivary gland [121,122] and midgut [106,123,124] of worker bees also secrete amylase. Thus, worker bees rely on their secreted amylase to hydrolyze starch into nutrients they can digest and absorb [118], providing flight energy [125].

In contrast, drones lack hypopharyngeal glands [107], and it is unclear if their midgut produces amylase, making it unknown whether they can digest starch. However, since drones are not involved in foraging or processing food [126], the absence of amylase may not negatively impact them. Drones obtain honey from honey cells and can digest starch even without secreting amylase. Catalase is a natural honey component [127,128], primarily derived from pollen, followed by nectar or yeast in honey [129]. Catalase regulates hydrogen peroxide balance, influencing glucose oxidase activity in honey [130] and antibacterial components [131]. Phosphatase in honey [132] comes from nectar, pollen, and bees [133], and its main function is to hydrolyze phosphate esters [134]. Glucose oxidase in honey, secreted by worker bees’ hypopharyngeal glands [135,136], mainly degrades glucose to gluconic acid by releasing small amounts of hydrogen peroxide [117,137,138].

Under active and passive evaporation, sucrose is hydrolyzed by invertase into fructose and glucose after bee colonies brew the sucrose solution for 48 h. Comparisons between bee species showed that the fructose and glucose contents of solutions processed by EHB colonies were significantly higher than those processed by WHB colonies. In contrast, the sucrose residual content in solutions processed by EHB colonies was significantly lower than in those processed by WHB colonies. This indicates that EHB invertase activity was higher than that of WHB, verified by the comparison of enzyme activities between species. Additionally, the total amylase and β-amylase activities in the sugar solution processed by EHB for 48 h were lower than those in the solution processed by WHB for 48 h, while the activities of the other six enzymes were higher in the EHB-processed solution. Enzyme activity is an indicator of honey quality [139] and is influenced by factors such as bee age, nectar abundance, and colony strength. As bees age, they transition from house bees to foraging bees, resulting in increased production of digestive enzymes [140,141,142], which elevates enzyme activity in honey. Nectar viscosity influences saliva secretion by bees, which, in turn, affects enzyme secretion [143,144]. The amount of saliva secreted into raw nectar or honeydew is mainly influenced by nectar flow and water content. High-concentration nectar or honeydew must be diluted with saliva, which increases enzyme activity in honey [143]. However, longer nectar flows and greater nectar quantity result in less saliva added to the raw material, lowering enzyme content in honey [145]. With large nectar flow and extended nectar flow duration, a significant amount of nectar is continuously brought back to the hive by forager bees, while house bees lack sufficient time to process the nectar quickly. The stronger the colony, the more worker bees contribute to nectar processing, leading to higher enzyme activity in honey [146,147].

## 5. Conclusions

This study indicates that neither the EHB nor the WHB prefers specific comb types for nectar storage. Enlarging cell diameter reduces nectar concentration efficiency in EHB colonies but enhances it in WHB colonies. The enzyme activity in ripe honey produced by EHB colonies is higher than that in ripe honey from WHB colonies, leading to a more efficient conversion of sucrose into monosaccharides. Nectar concentration increases with longer passive evaporation time. The nectar concentration efficiency of WHB colonies is higher than that of EHB colonies due to their distinct ventilation strategies.

## Data Availability

The preprocessed data that support the findings of this study are available from the corresponding author upon reasonable request.
